# Clinical, laboratory, and procedural predictors of slow flow/no reflow phenomenon after emergency percutaneous coronary interventions in ST-elevated myocardial infarction

**DOI:** 10.1186/s43044-024-00577-0

**Published:** 2024-11-04

**Authors:** Fatemeh Bamarinejad, Mohammad Kermani-alghoraishi, Azam Soleimani, Hamidreza Roohafza, Safoura Yazdekhasti, Maedeh Azarm, Atefeh Bamarinejad, Masoumeh Sadeghi

**Affiliations:** 1 Isfahan Cardiovascular Research Center, Cardiovascular Research Institute, Isfahan University of Medical Sciences, Isfahan, Iran; 2 Interventional Cardiology Research Center, Cardiovascular Research Institute, Isfahan University of Medical Sciences, Isfahan, Iran; 3 Heart Failure Research Center, Cardiovascular Research Institute, Isfahan University of Medical Sciences, Isfahan, Iran; 4 Cardiac Rehabilitation Research Center, Cardiovascular Research Institute, Isfahan University of Medical Sciences, Isfahan, Iran; 5 Department of Cardiology, Chamran Cardiovascular Medical and Research Hospital, Isfahan University of Medical Sciences, Isfahan, Iran

**Keywords:** ST-elevated myocardial infarction, Percutaneous coronary intervention, Coronary slow flow phenomenon, No reflow, Predictor

## Abstract

**Background:**

Emergency percutaneous coronary intervention (PCI) is a common treatment for ST-elevated myocardial infarction (STEMI) patients. However, the coronary slow flow/no reflow phenomenon (CSF/NRP) can occur as a complication during or after the procedure. Identifying predictors of CSF/NRP after emergency PCI in STEMI patients can help clinicians anticipate and prevent this complication. In this study, we aimed to investigate clinical, laboratory, and procedural factors that may contribute to the development of CSF/NRP in STEMI patients undergoing PCI.

**Results:**

A total of 460 patients were included in this study, with a mean (± SD) age of 60 ± 12.5 years. The incidence of CSF/NRP was 30.2% (n = 139) among the study population. The univariate analysis showed that older age, lower left ventricular ejection fraction (LVEF), initial thrombolysis in myocardial infarction (TIMI)flow grade 0–2, increased creatinine level, lower estimated glomerular filtration rate (eGFR), diffuse target lesion length, and longer length of stent were significantly associated with the occurrence of CSF/NRP (p < 0.05). However, in the multivariate logistic regression model, only eGFR (OR = 0.98, 95% CI: 0.96–0.99, p = 0.005), diffuse target lesion length (OR = 2.15, 95% CI: 1.20–3.83, p = 0.009) and LVEF (OR = 0.96, 95% CI: 0.94–0.98, p = 0.004) remained significant predictors of CSF/NRP.

**Conclusions:**

The present study demonstrated that diffuse lesion length, lower LVEF, and lower eGFR can be considered as independent predictors of CSF/NRP in STEMI patients.

## Background

The coronary slow flow phenomenon (CSFP) is an angiographic finding that refers to the slow antegrade passage of contrast agents through distal segments of epicardial coronary arteries without substantial stenosis [1, 2]. CSFP appears to have a multifaceted pathophysiology, and variables such as functional and morphological impairments of the microvasculature, concealed atherosclerosis, the inflammatory process, and ischemic injury may all contribute to its development [3, 4].

The occurrence of this phenomenon among individuals who have diagnostic angiography ranges from 1.5 to 5.5% [5–8]. However, in more than 30% of STEMI patients who have undergone PCI, the coronary slow flow/no reflow phenomenon (CSF/NRP) leads to inadequate myocardial reperfusion despite the presence of fully patent coronary arteries [9–12]. Additionally, STEMI patients who develop CSF/NRP are at a heightened risk for mortality, along with the occurrence of left ventricular dysfunction and further advancement of myocardial damage [13, 14].

A network meta-analysis article suggests that intracoronary use of adenosine can improve clinical outcomes in STEMI patients undergoing PCI and reduce the incidence of CSF/NRP[15]. The use of such pharmacologic agents for individuals at increased risk of post-PCI CSF/NRP seems reasonable. Therefore, pre-procedural risk assessment of CSF/NRP enables timely intervention and preventive initiation of a therapeutic approach that minimizes the occurrence of no-reflow.

The consideration of clinical, laboratory, and procedural parameters prior to emergent PCI can provide valuable insights for predicting the incidence of CSF/NRP in STEMI patients. A systematic review and meta-analysis study has introduced initial thrombolysis in myocardial infarction (TIMI) flow and high thrombus burden as strong risk factors for CSF/NRP in STEMI patients, but it also points out the need for more studies with a larger sample size [16]. The current investigation was conducted to determine clinical and laboratory variables as well as procedural characteristics that predict the no reflow phenomenon in STEMI patients following emergent PCI.

## Methods

### Study design and population

The current study is a secondary analysis of data from the early phase (I/II) of the SEMI-CI study [17]. From March 2015 to March 2016, eligible patients who were diagnosed with acute STEMI and subsequently underwent primary or rescue PCI were included in this study. We applied the international definition for STEMI diagnosis. This study excluded patients with missing angiographic data.

The Isfahan University of Medical Sciences' Ethical Review Committee approved the current study (Ethical approval number: IR.MUI.MED.REC.1400.601). Informed consent was obtained from all the participants or their first relatives. To maintain confidentiality, the patient data were encoded and made anonymous.

### Revascularization procedures

Oral aspirin (300 mg) and clopidogrel (300 mg), in addition to intravenous 5000–10000 U unfractionated heparin, were administered to all patients prior to the procedure. Coronary angiographies were performed through the femoral or radial artery to determine the culprit lesion, and then, percutaneous coronary intervention was performed as reperfusion therapy. According to the coronary angiography features, GP IIb/IIIa inhibitors and/or aspiration catheters were used. The decision on the administration of balloon angioplasty or stent placement was made by the cardiologist based on target lesion characteristics. Drug-eluting stents were mostly used for stenting procedures.

Offline analysis of digital angiograms retrospectively was performed by two experienced cardiologists who were blinded to the clinical data. The initial and post-procedural blood flow in the infarct-related artery was graded according to the TIMI grading system [18]. The end-procedural TIMI grades 0, 1, and 2 flow despite successful dilatation and absence of mechanical complications such as dissection, spasm, or angiographically evident distal embolization were described as a “slow flow/no reflow” phenomenon.

### Data extraction

The study was retrospective, and demographic, clinical, and laboratory data were acquired from patient history files. According to the protocol of our center, blood samples for complete blood count, glucose, renal functions, coagulation test, electrolytes, and qualitative cardiac biomarkers of STEMI patients were routinely sent before angiography. Fasting blood sugar, lipid profiles, and other tests, if needed, were measured after 24 h from admission to emergency service at the intensive care unit. Glomerular filtration rate (GFR) was measured by creatinine clearance according to the Cockcroft-Gault formula (19). The eGFR was adjusted using the "Du Bois and Du Bois" formula for body surface area [20].

Angiographic data were extracted from PCI reports. These parameters included: method of PCI, types of total occlusion if present (subtotal, tapered, or cut-off lesion), lesion location (proximal, mid, or distal lesion), length of the target lesion (discrete, tubular, or diffuse), and the features of the stent if used.

### Statistical analysis

We first used descriptive analyses to compare demographic and clinical characteristics between no reflow and normal flow patients. We present categorical data with frequencies and percentages and continuous data with a mean (± standard deviation [SD]) or median (interquartile range [IQR]). The normality of all continuous variables was checked using the Kolmogorov–Smirnov test. For descriptive statistics, we used the χ2 test for categorical variables and the independent samples t test or Mann–Whitney U test for continuous variables. Potential predictive factors of CSF/NRP were considered in the statistical analysis. Univariate analysis and then forward conditional multivariate logistic regression analysis were used to identify the risk factors of no reflow. The significance level was established as P values of 0.05. Statistical analysis was performed using IBM SPSS Statistics, version 26.0 (IBM Corp., Armonk, NY, USA).

## Results

### Baseline characteristics

A total of 666 patients diagnosed with STEMI underwent emergent PCI in our hospital during 2015–2016. Among them, 206 patients were excluded from the study due to missing key laboratory or procedural data. The study enrolled 460 patients (83.3% male) with a mean age of 60 ± 12.5 years. Angiographic no reflow was seen in 139 (30.2%) patients at the end of PCI, including 116 males and 23 females, with a mean age of 62.5 ± 12.2 years. Therefore, the incidence of no reflow was 30.2%.

Baseline characteristics and demographic data are presented in Table 1. The no reflow group was significantly older than the normal flow group (62.58 ± 12.25 vs. 58.88 ± 12.53 years, P = 0.002). However, none of the additional baseline variables demonstrated statistically significant variations between the two groups.

**Table 1:** Demographic characteristics of the study patients in reflow and no reflow group

Variables	Total N = 460	Normal flow TIMI 3 N = 321(69.8%)	No Reflow TIMI 0–2 N = 139(30.2%)	P value
Age, *year(mean* ± *SD)*	60 ± 12.5	58.88 ± 12.53	62.58 ± 12.25	0.002*
Male gender	383 (83.3%)	267(83.1%)	116(83.4%)	0.94
Diabetes mellitus	135(29.3%)	94(29.2%)	41(29.4%)	0.98
Hypertension	156(33.9%)	110(34.2%)	46(33%)	0.86
Peripheral artery disease	17(3.6%)	13(4%)	4(2.8%)	0.80
Current smoker	196(42.6%)	146(45.4%)	50(35.9%)	0.063
Body mass index, *kg/m*^*2*^	26.19 ± 4.17	26.25 ± 4.15	26.03 ± 4.23	0.63
Previous CVD history				
Prior stroke/TIA	26(5.6%)	16(4.9%)	10(7.1%)	0.63
Prior MI	56(12.1%)	43(13.3%)	13(9.3%)	0.27
Prior PCI	46(10%)	32(9.9%)	14(10%)	0.931
Prior CABG	9(1.9%)	5(1.5%)	4(2.8%)	0.62
Previous medication				
Aspirin	155(33.6%)	112(34.8%)	43(30.9%)	0.41
P2Y12 inhibitors	22(4.7%)	19(5.9%)	3(2.1%)	0.083
Beta blockers	86(18.6%)	56(17.4%)	30(21.5%)	0.29
Statins	107(23.2%)	79(24.6%)	28(20.1%)	0.29
ACEI/ARB	91(19.7%)	60(18.6%)	31(22.3%)	0.37
MRA	4(0.8%)	3(0.9%)	1(0.7%)	0.64
Diuretics	17(3.6%)	12(3.7%)	5(3.5%)	0.94

TIMI: Thrombolysis in Myocardial Infarction, TIA: Transient ischemic attack, MI: Myocardial infarction, PCI: Percutaneous coronary intervention, CABG: coronary artery bypass grafting, ACEI: angiotensin-converting enzyme inhibitor, ARB: angiotensin receptor blocker, MRA: Mineralocorticoid receptor antagonist. *Significant difference at the 0.05 level

### Clinical and laboratory characteristics

Patients with CSF/NRP had significantly reduced LV function compared to those in the normal flow group. In keeping with this, a numerical trend was evident for the higher Killip class in the NR group; however, this did not reach statistical importance (p = 0.502). Also, the level of HDL in this group was significantly lower than in normal flow patients. In addition, in CSF/NRP patients, creatinine levels were significantly higher and eGFR was significantly lower than in the normal flow group (65.55 ± 20.68 vs. 72.39 ± 20.36 ml/min/1.73 m^2^, p = 0.003). Tables 2 and 3 show the clinical and laboratory variables in two groups, respectively.

**Table 2:** Clinical data characteristics of the study patients in normal flow and no reflow group

Variables	Total N = 460	Normal flow TIMI 3 N = 321(69.8%)	No reflow TIMI 0–2 N = 139(30.2%)	P value
Ventilation on admission, *n(%)*	32(6.9%)	7(2.1%)	25(17.9%)	0.28
Admission SBP, *mmHg (mean* ± *SD)*	126.39 ± 25.83	127.41 ± 26.87	126.3 ± 25.2	0.77
Admission PR, beat/min* (mean* ± *SD)*	79 ± 22.18	80.93 ± 22.48	77.84 ± 21.11	0.36
ECG MI status				
Anterior, *n(%)*	249(54.1%)	166(51.7%)	83(59.7%)	0.11
Non-anterior, *n(%)*	211(45.8%)	155(48.2%)	56(40.2%)	
Symptom onset to PCI, *Hour (mean* ± *SD)*	7.52 ± 14.78	7.81 ± 17.34	6.87 ± 5.56	0.56
LVEF, %(*mean* ± *SD*)	37.755 ± 11.62	39.25 ± 11.70	34.03 ± 10.59	< 0.001
Emergency PCI status				
Primary PCI, *n(%)*	261(56.7%)	178(55.4%)	83(59.7%)	0.39
Rescue PCI, *n(%)*	199(43.2%)	143(44.5%)	56(40.2%)	
Admission Killip class				
I	427(92.8%)	299(93.1%)	128(92%)	0.79
II	25(5.4%)	17(5.2%)	8(5.7%)	
III	1(0.2%)	1(0.3%)	0(0%)	
IV	7(1.5%)	4(1.2%)	3(2.1%)	

SBP: Systolic Blood Pressure; PR: Pulse Rate. *Significant difference at the 0.05 level

**Table 3:** Laboratory data of the study patients in normal flow and no reflow group

Variables	Total N = 460	Normal flow TIMI 3 N = 321(69.8%)	No reflow TIMI 0–2 N = 139(30.2%)	P value
Hemoglobin level, *g/dl*	14.34 ± 1.8	14.93 ± 1.12	13.45 ± 2.17	0.78
Hematocrit, *%*	42.71 ± 6.4	42.66 ± 6.6	42.8 ± 5.93	0.83
White blood cell count, × *10*^*9*^* /L*	12,529 ± 10,475	18,400 ± 23,136	10,700 ± 5207	0.57
Platelet count, × *10*^*3*^* /mL*	205.79 ± 67.01	210.74 ± 41.55	195.88 ± 47.21	0.38
Neutrophil count, */mL*	9754 ± 8849	10,669 ± 11,261	10,055 ± 4780	0.46
Lymphocyte count, */mL*	2024 ± 2256	24.01 ± 3064	1716.38 ± 761.93	0.20
Monocyte count, */mL*	731.77 ± 622.83	748.27 ± 688.93	693.00 ± 429.24	0.22
Neutrophils-to- lymphocyte ratio	6.70 ± 5.34	6.38 ± 4.58	6.95 ± 4.13	0.63
Platelet-to-lymphocyte ratio	137.8 ± 79.35	124 ± 74.68	149.9 ± 77.07	0.11
Lymphocyte-to-monocyte ratio	3.69 ± 3.55	3.74 ± 3.75	3.57 ± 3.02	0.45
Red cell distribution width, *%*	13.52 ± 1.49	12.5 ± 2.32	14.8 ± 3.12	0.69
RDW-platelet ratio × 10^–3^	0.072 ± 0.023	0.07 ± 0.02	0.06 ± 0.01	0.27
Glucose plasma level, *mg/dl*	172.8 ± 84.15	169.95 ± 77.46	167.13 ± 54.96	0.14
Fasting blood sugar, *mg/dl*	125.7 ± 47.93	115.74 ± 40.92	124.75 ± 39.48	0.43
Total cholesterol, *mg/dl*	165.48 ± 59.72	156.84 ± 40.37	143.5 ± 66.66	0.57
Triglyceride, *mg/dl*	155.25 ± 89.87	161.19 ± 96.31	141.12 ± 70.86	0.10
LDL-C, *mg/dl*	106.23 ± 37.28	111.84 ± 37	105.75 ± 40.96	0.91
HDL-C, *mg/dl*	38.68 ± 9.66	41.89 ± 10.61	35.00 ± 8.48	0.047*
Creatinine level, *mg/dl*	1.19 ± 0.31	1.06 ± 0.17	1.14 ± 0.25	0.049*
Blood urea nitrogen, *mmol/L*	37.73 ± 19.48	36.42 ± 14.33	38.38 ± 14.04	0.32
eGFR, ml/min/1.73m^2^	70.43 ± 20.66	72.39 ± 20.36	65.55 ± 20.68	0.003*
Serum Na^+^, *mEq/L*	142.17 ± 4.18	141.4 ± 3.74	142.88 ± 3.04	0.93
Serum K^+^, *mEq/L*	4.44 ± 0.51	4.38 ± 0.5	4.45 ± 0.42	0.48
Prothrombin time, *s*	19.10 ± 10.87	25 ± 15.1	17.43 ± 8.35	0.32
International normalized ratio (INR)	1.58 ± 1.23	1.58 ± 1.28	1.58 ± 1.12	0.98
Partial thromboplastin time, *s*	38.09 ± 19.61	45 ± 27.56	34.38 ± 16.82	0.44
Aspartate aminotransferase, *U/L*	133.45 ± 134.9	143.21 ± 134.5	125.75 ± 171.9	0.92
Alanine aminotransferase, *U/L*	59.34 ± 64.42	53.73 ± 28.51	36.38 ± 31.99	0.79
Alkaline phosphatase, *U/L*	209.88 ± 67.45	209.1 ± 50.02	270.75 ± 135.27	0.02*
AST/ALT Ratio	2.48 ± 1.6	2.77 ± 1.73	2.57 ± 1.58	0.34

RDW: Red cell distribution width, LDL-C: Low-density lipoprotein-cholesterol, HDL-C: High-density lipoprotein-cholesterol, eGFR: estimated glomerular filtration rate. *Significant difference at the 0.05 level

### Coronary angiographic characteristics

**Fig. 1:**
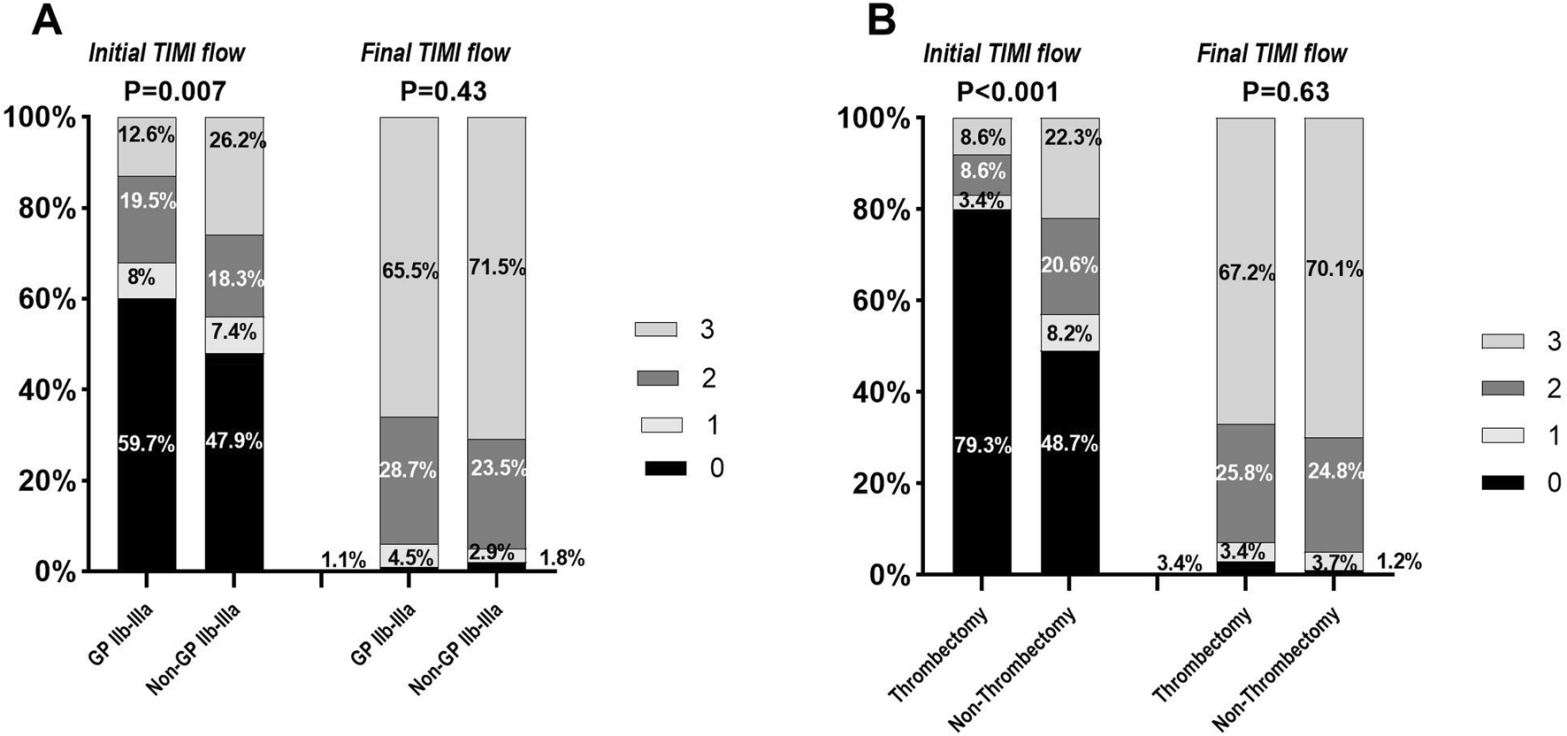
Initial and final TIMI flow grade according to the use or not of GP IIb/IIIa inhibitors (**A**) and intracoronary aspiration thrombectomy (**B**)

Angiographic and procedural parameters are listed in Table 4. A higher percentage of CSF/NRP patients had an initial TIMI less than 3 compared to the normal flow group (89.9% vs. 74.7%, p < 0.001). There was also a significant difference in the length of the lesion between the two groups. Subsequently, the stent length in the CSF/NRP group was significantly longer than those in the normal flow group (33.98 ± 18.12 vs. 29.27 ± 15.10mm, p = 0.005). Our findings indicate that preventive measures, such as the use of GP IIb/IIIa inhibitors and aspiration thrombectomy, were implemented more in high thrombus burden group than in the non-high thrombus burden group (GP IIb/IIIa: 78% vs. 30.8%, p < 0.001؛ thrombectomy: 54.8% vs. 2.5%, p < 0.001). The initial TIMI flow differed significantly between the group received GP IIb-IIIa inhibitors and the group not received these agents, with a notably higher percentage of initial TIMI 0 flow observed in the GP IIb-IIIa group (59.7% vs. 47.9%, p = 0.007). However, upon evaluating the final TIMI flow, no significant differences were found between the two groups. This finding suggests that the administration of GP IIb-IIIa inhibitors may effectively enhance final TIMI flow outcomes. Similar trends were noted when comparing the differences in initial and final TIMI flow rates within both thrombectomy and non-thrombectomy cohorts (Fig. 1).

**Table 4:** Procedural characteristics of the study patients in normal flow and no reflow group

Variables	Total N = 460	Normal flow TIMI 3 N = 321(69.8%)	No reflow TIMI 0–2 N = 139(30.2%)	P value
Initial TIMI flow				
0–2	365(77.3%)	240 (74.7%)	125(89.9%)	< 0.001
3	95(20.6%)	81(25.2%)	14(10%)	
Infarct-related coronary artery				
LM	3(0.6%)	3(0.9%)	0(0%)	0.29
LAD/diagonal	254(55.2%)	169(52.6%)	85(61.1%)	
LCX/OM	44(9.5%)	32(9.9%)	12(8.6%)	
RCA	155(33.6%)	115(35.8%)	40(28.7%)	
PDA/PLV (others/SVG)	4(0.8%)	2(0.6%)	2(1.4%)	
Target lesion location				
Proximal	244(53%)	168(52.3%)	76(54.6%)	0.30
Mid	159(34.6%)	106(33%)	53(38.1%)	
Distal	35(7.6%)	28(8.7%)	7(5%)	
Type of occlusion				
Cut off	212(46.1%)	140(43.6%)	72(51.7%)	0.37
Subtotal	99(21.5%)	71(22.1%)	28(20%)	
Tapered	131 (28.5%)	95(29.5%)	36(25.8%)	
Lesion length				
Discrete	36(7.8%)	24(7.4%)	12(8.6%)	0.018*
Tubular	126(27.4%)	100(31.1%)	26(18.7%)	
Diffuse	266(57.8%)	174(52.2%)	92(66.1%)	
High thrombus burden	82(17.8%)	57(17.7%)	25(17.9%)	0.52
GP IIb-IIIa use	174(37.8%)	114(35.5%)	60(43.1%)	0.18
Aspiration thrombectomy	58(12.6%)	39(12.1%)	19(13.6%)	0.65
Hemodynamic support	26(5.6%)	11(3.4%)	16(11.5%)	0.22
Method of PCI				
POBA	54(11.7%)	32(9.9%)	22(15.8%)	0.28
Predilation + Stenting	291(63.3%)	204(63.5%)	87(62.5%)	
Predilation + Stenting + Postdilation	20(4.3%)	17(5.2%)	3(2.1%)	
Stenting + Postdilation	9(2%)	6(1.8%)	3(2.1%)	
Direct stenting	66(14.3%)	46(14.3%)	20(14.3%)	
Mean stent length	30.65 ± 16.12	29.27 ± 15.10	33.98 ± 18.12	0.005*
Mean Stent diameter	3.08 ± 0.38	3.06 ± 0.4	3.1 ± 0.37	0.26
Multiple stent (≥ 2 stent)	122(26.5%)	82(25.5%)	40(28.7%)	0.57
In-stent thrombosis	42(9.1%)	31(9.6%)	11(7.9%)	0.52

LAD: Left anterior descending, RCA: Right coronary artery, LCX: Left circumflex, OM: Obtuse marginal, POBA: Plain old balloon angioplasty. *Significant difference at the 0.05 level

### Independent Predictors of the CSF/NRP

We performed univariate and then multivariate binary logistic regression analyses to find the independent predictors of CSF/NRP. Table 5 highlights the independent predictors of angiographic slow flow/no reflow. Univariate regression analyses showed that older age,

**Table 5:** Univariate and multivariate logistic regression analysis to determine independent predictors of coronary slow flow/no reflow

Variables	Univariate			Multivariate		
	OR	CI	P-value	OR	CI	P value
Age	1.024	1.008–1.041	0.004	−	−	−
Initial TIMI flow	3.01	1.64–5.53	< 0.001	−	−	−
LVEF	0.96	0.94–0.98	< 0.001	0.98	0.96–0.99	0.004
Mean stent length	1.017	1.005–1.03	0.008	−	−	−
Lesion length (Diffuse)	1.72	1.10–2.68	0.016	2.15	1.20–3.83	0.009
eGFR	0.98	0.97–0.99	0.003	0.96	0.94–0.98	0.005
Creatinine level	2.03	1.08–3.81	0.028	−	−	−

TIMI: Thrombolysis in Myocardial Infarction, LVEF: Left ventricular ejection fraction, eGFR: estimated glomerular filtration rate, OR: Odds ratio, CI: Confidence interval

initial TIMI flow less than 3, lower LVEF, higher lesion length (diffuse), higher stent length, higher creatinine level, and lower eGFR were correlated with the incidence of CSF/NRP. However, only lesion length, LVEF, and eGFR remained significant after multivariate regression.

## Discussion

In this study, we found a prevalence of 30 percent of post-procedural CSF/NRP in STEMI patients. Our study revealed that lower left ventricular function, lower eGFR, and diffuse lesion length are the independent predictors of CSF/NRP among STEMI patients treated with emergent PCI. Moreover, there was a positive correlation between older age, initial TIMI flows less than 3, increased creatinine level, longer stent length in STEMI patients, and higher odds of developing CSF/NRP.

To date, several investigations have been performed to identify predictors of CSF/NRP, and several clinical and procedural features have been proposed and found to be predictive [10, 21–24]. Regarding patients diagnosed with STEMI, a systematic review and meta-analysis of the existing literature has been conducted on a total of 27 studies to identify potential predictors of the post-PCI slow flow phenomenon. However, they pointed out that their analysis just focused on the covariates investigated within the selected studies without considering additional factors [16]. In the present investigation, we attempted to assess the impact of additional variables on the development of CSF/NRP in patients diagnosed with STEMI.

No reflow is currently recognized as a dynamic process that is caused by the interaction of multiple mechanisms, particularly preexistent endothelial dysfunction, distal atherothrombotic embolization, ischemia/reperfusion injury, and individual susceptibility [3]. Age-related pathologic changes in vascular structures, including preexistent microvascular and endothelial dysfunction, vascular calcification, and stiffness, along with altered neurohormonal and autonomic effects, have the potential to contribute to microvascular obstruction and distal embolization during PCI, thereby giving rise to the no reflow phenomenon [25, 26]. The results of the present study integrated into the literature demonstrate a correlation between advancing age and a diagnosis of no reflow [16, 27–29].

The effective use of risk estimation and categorization before intervention might provide beneficial guidance to interventional cardiologists to prevent the occurrence of no reflow as well as enable more timely management if no reflow does occur [30]. Several clinical studies during the past decade have demonstrated a significant correlation between some angiographic characteristics and the no reflow phenomenon [16, 23]. In line with previous studies, our results revealed that angiographic and procedural parameters such as initial TIMI flow, lesion length, and mean stent length were associated with no reflow. In particular, the diffuse length of the target lesion has been identified as an independent predictor for the development of CSF/NRP. A greater length of the lesion impacts the flow restoration and raises the risk of CSF/NRP, according to findings by other studies conducted in various contexts [10, 29, 31–33], even if some of the prior investigators have not considered the effect of target lesion length in their studies.

As the length of a target lesion increases, it is associated with an increased accumulation of plaque and thrombus burden [34, 35]. Also, the reactivity of the arteries declines, and the capacity for compensatory endothelial vasodilatation of the vessels becomes limited [3, 32, 36]. Moreover, the use of extended stents in such situations has been correlated with plaque prolapse and myonecrosis after stenting [37, 38], which ultimately can increase the risk of post-PCI CSF/NRP.

There seems to be a positive correlation between the length of the lesion and the thrombus burden. Despite there being an association between the lesion length and the incidence of CSF/NRP, we failed to identify a correlation between thrombus burden and the occurrence of CSF/NRP. Our findings indicate that preventive measures, such as the use of GP IIb/IIIa inhibitors and aspiration thrombectomy, were particularly implemented in cases with high thrombus burden. Although our results suggest that these proactive measures improve post-PCI TIMI flow and reduce the occurrence of CSF/NRP, the lack of a direct correlation between thrombus burden and CSF/NRP occurrence may be attributed to these management strategies. Greenland et al. reported a significant relationship between thrombus burden and CSF/NRP, regardless of the lesion length [39]. Additionally, a meta-analysis conducted by Fajar et al. noted both thrombus burden and lesion length as independent predictors of CSF/NRP [16].

Although some studies have introduced risk scores for the occurrence of CSF/NRP in STEMI patients, it seems that laboratory factors have a lesser place in these risk scores [40, 41]. Kumar et al. introduce a risk stratification model for slow flow/no reflow during primary percutaneous coronary intervention (the RK-SF/NR Score), which includes contextual, clinical, and angiographic variables [33], while, in our study, the creatinine level (as a laboratory predictor) and, consequently, the eGFR are factors associated with the incidence of CSF/NRP in STEMI patients.

It is well known that chronic kidney disease is a risk factor for cardiovascular diseases [42]. In addition, severe renal dysfunction seems to cause disturbances in coronary blood flow through factors such as ventricular hypertrophy or coronary epicardial artery stenosis [43]. Along with our study, Kurtul et al. showed that impaired renal function is associated with an increased risk of CSF/NRP in patients who underwent primary PCI. However, they excluded patients with severe renal dysfunction due to the potential effects it could have on coronary blood flow [44]. In another study, Kai et al., by limiting the samples to STEMI patients with an initial TIMI flow equal to 0, found similar results [21]. However, the correlation between no reflow and renal function in NSTEMI patients has also been observed; the decrease in eGFR has been associated with an increase in the probability of no reflow compared to coronary slow flow (45).

There were several limitations that should be considered when interpreting our results. First of all, the retrospective nature of our study may have led to selection biases and information biases. This was a registry-based study with a relatively small sample size, so even though a multivariate model was applied to control for potential sources of error, there are still bound to be some. Further studies with larger sample sizes and more comprehensive data collection methods are needed to confirm our findings.

## Conclusions

In conclusion, the present study demonstrated that lower eGFR, reduced LVEF, and diffuse length of the lesion are independent predictors of CSF/NRP in STEMI patients undergoing emergent PCI. These findings highlight the importance of careful pre-procedural risk assessment to identify high-risk patients for CSF/NRP and initiate preventive measures to minimize its occurrence. Further studies with larger sample sizes are needed to confirm these findings and develop a more comprehensive risk prediction model for CSFP in STEMI patients.

## Data Availability

The datasets used during the current study are available from the corresponding author upon reasonable request.

## Abbreviations

CSF/NRP: Coronary slow flow/no reflow phenomenon
eGFR: Estimated glomerular filtration rate
IRA: Infarct-related artery
LVEF: Left ventricular ejection fraction
PCI: Percutaneous coronary intervention
STEMI: ST-elevated myocardial infarction
TIMI: Thrombolysis in myocardial infarction
